# Vibration Energy Conversion Power Supply Based on the Piezoelectric Thin Film Planar Array

**DOI:** 10.3390/s22218506

**Published:** 2022-11-04

**Authors:** Bo Wang, Dun Lan, Fanyang Zeng, Wei Li

**Affiliations:** State Key Laboratory of Functional Materials for Informatics, Shanghai Institute of Microsystem and Information Technology, Chinese Academy of Sciences, Shanghai 200050, China

**Keywords:** vibration energy harvesting, energy harvesting interface circuit, energy scavenging, battery-less, self-powered power supply, ambient power, PVDF piezoelectric material

## Abstract

Vibration energy harvesting has received much attention as a new type of power solution for low-power micro/nano-devices. However, VEH (vibration energy harvester) based on PVDF (polyvinylidene fluoride) piezoelectric materials have a low output power and energy conversation efficiency due to the relatively low piezoelectric constant, coupling coefficient, and dielectric constant. For this reason, we design a vibration energy conversion power supply, which consists of a VEH with a PVDF piezoelectric thin film planar array vibration structure and an energy harvesting circuit for regulating the electric energy of multiple sources. Furthermore, our solution was validated by simulations of structural dynamics in COMSOL and equivalent circuits model in Multisim. From the circuitry simulation results, the output current and the charging period increase and decrease, doubling, respectively, for each doubling of the number of array groups of films. Moreover, the solid mechanics simulation results show that the planar array structure makes the phase and amplitude of the input vibration waves as consistent as possible so that the same theoretical enhancement effect of the circuitry model is achieved. An identical experimental test was implemented with vibration conditions of 75 Hz-2.198 g. The fabricated harvester quickly charged the 22 V-0.022 F ultracapacitor bank to 5 V in 24 min. The maximum open circuit voltage and output power, respectively, were 10.4 V and 0.304 mW. This maximum charging power was 11.69 times higher than that of a single film. This special power supply can replace batteries to power low-power electronics deployed in vibrating environments, thus reducing the maintenance costs of equipment and environmental pollution rates.

## 1. Introduction

With the development of the technology of IoT (internet of things), electronic devices are increasingly common everywhere, in both daily life and industry. Regardless of whether the devices are single-structure devices or parts of a multi-functional system, it needs a power supply [1]. These devices are not always powered by power lines and are often equipped with batteries, but battery-driven smart terminals are very limited in power, thus reducing the equipment s’ reliability. In addition, a large amount of electronic equipment increases the system power supply module design complexity, equipment costs, and maintenance costs. Taking wireless sensor networks as an example, the size and weight of each node limit long-term energy storage, and the battery maintenance costs are too high because of the large number of deployed nodes. Sometimes, the deployment location or packaging of wireless sensor nodes is very special; hence, the maintenance of cell modules is considered impractical [2].

Based on the present situation, energy harvesting technology has emerged. It is a technology that uses various new energy exchange materials, structures, or systems to convert unused environmental energy into electric energy for storage and utilization. Potential energy sources are solar, wind, thermal electric, biomass, chemical, vibrational, etc. [3]. Among them, vibration energy is widely available and easy to harvest and is a green energy source. It is usually preferred because of the high output voltage, high power density, and simple configuration of the device. The method of acquiring vibrational energy can be divided into electromagnetic, electrostatic, piezoelectric, magneto-strictive, etc. In recent years, the benefit of the universality of piezoelectric materials in sensing applications, such as health monitoring, fault identification, and non-destructive testing [4,5,6], research on piezoelectric vibration energy harvesting technology has made great progress and garnered wide attention in vibrational structures, energy harvesting circuits, and so on [7]. Some piezoelectric materials with great electromechanical coupling characteristics not only be used for sensing and detection but also be used for the main component of the piezoelectric energy harvester for generating power. Piezoelectric ceramic materials such as PZT are widely used piezoelectric materials with mature technology and low cost. Piezoelectric ceramic material has the characteristics of large piezoelectric coefficients and high vibration sensitivity and energy transfer efficiency; hence, it is suitable for the fabrication of transducers for harvesting vibration energy. Kuo et al. [8] suggested the deposition of PZT layers on both sides of a stainless steel substrate using a customized aerosol deposition machine to fabricate an energy harvesting device. The cantilever harvester with the technique was tested under a base excitation of 1.5 g at 140.8 Hz, and a power output of 413 μW was measured. Tian et al. [9] presented a low-frequency MEMS piezoelectric energy harvester consisting of a PZT thin film on a flexible phosphor bronze substrate with a proof mass. A rectangular hole was created on the beam harvester to reduce the resonance frequency of the system. Exciting the device under a base acceleration of 1.5 g at 34.3 Hz, a power output of 216.66 μW was collected for an optimally matched load resistance of 60 kΩ. Shuibao Qi et al. [10] report on an innovative and practical acoustic energy harvester based on a defected acoustic metamaterial (AMM) with PZT piezoelectric material. The maximum output voltage and power of 1.3 V and 8.8 μW are acquired with an acoustic incidence of 2 Pa at a frequency of 2257.5 Hz. The literature [11] summarizes some related studies on mechanical energy harvesters that use mechanical metamaterials or phononic crystals and PZT material in combination. The output power and voltage of piezoelectric harvesting devices mentioned in this literature are not high. Most of the power is less than 100 μW, and only a few can reach 1000 mW, but the output voltage cannot exceed 5 V. However, the piezoelectric ceramic material is not flexible enough and is brittle [12], and the lifetime and reliability of the VEH are still affected by the brittleness of the material. Therefore, harvesters have certain application limitations in high-frequency, wide-frequency, and large-amplitude vibrational environments.

Compared with piezoelectric ceramic materials, PVDF materials are characterized by good frequency adaptability, flexibility, and small electrical impedance. Therefore, its service life is long and more suitable for vibration energy harvesting in the above vibrational environments. However, harvesters based on PVDF have a low output power, energy conversion efficiency, and material utilization rate and possess poor adhesion to the material because their coupling coefficient, dielectric and piezoelectric properties are slightly unsatisfactory for generating power [13]. Consequently, improving the output characteristics of these harvesters has become one of the design priorities. Shivaji H. Wankhade et al. [12] prepared a nanohybrid of PVDF and PZT to fabricate harvesters to convert waste mechanical energy from various human motions to useful electrical energy. The device with a size of 20 × 9 × 0.18 mm^3^ was able to generate a maximum output voltage of 55 V and a maximum power of 64.8 μW. In the same year, to improve the output characteristics of a single PVDF piezoelectric cantilever beam generator, a design that changes the stiffness of the cantilever beam was studied by Y. W. NING et al. [14]. A PVDF cantilever piezoelectric wind energy generator based on the results of the optimal design was established. When the wind speed was in the range of 4~14 m/s, the device with dimensions of 79.86 × 19.67 × 0.12 mm^3^ had a maximum output voltage of 18.6 V and a maximum power of 1.3 μW. In addition, researchers from Japan successively made a bimorph piezoelectric vibration energy harvester with a flexible 3D meshed-core elastic layer to improve the output power while lowering the resonance frequency. A harvester with dimensions of 10 × 20 × 0.3 mm^3^ generated a maximum output voltage of 20 V and a maximum power of 24.6 μW [15].

The above existing studies indicate that the main current solutions include improved materials, vibration structures, and harvesting circuits. However, the improvement of the output characteristics, such as voltage and power of these harvesters, is less obvious. To further improve the power generation capacity and the environmental adaptability of the piezoelectric VEH and to make it more practical, i.e., combining the merits of both PZT and PVDF, we fabricate a PVDF thin film with smaller dimensions (46.4 × 13.21 × 0.4 mm^3^) in this letter, and design a vibration energy conversion power supply that consists of a vibrational structure of the thin film planar array and an electric energy regulation circuit for paralleling multiple current sources input. We take the range of vibrational frequencies of 0~200 Hz, which is common in daily life and engineering, as the experimental conditions [16]. The solution in this paper was validated by simulations of structural dynamics and equivalent circuits, and it improves the energy conversion efficiency and charging rate of the whole device. In the experimental tests, the power supply module charges at 22 V-0.022 F ultracapacitor bank to 5 V within half an hour and outputs a standard voltage of 3.6 V at the output terminal. It also has an open circuit voltage of 10.4 V and a maximum power of 0.304 mW, which is 11.69 times higher than that of a single film. The stored electric energy is able to light an LED of 60 mW or maintain a Bluetooth RF device of 3 mW for a period of time.

## 2. Energy Conversion Principle and Circuit Model of Vibration Energy Harvester

The principle of power generation of piezoelectric materials is the piezoelectric phenomenon [17]. When the material is continuously compressed and stretched, an AC voltage appears between the electrodes, and AC power is output in the rear circuit. The mechanical structure of VEH can capture ambient vibration to occur displacement or deformation, which the piezo-material needs. Cantilever beams, simply supported beams, rectangular beams, and circular and cymbal structures are common [18].

**Figure 1:**
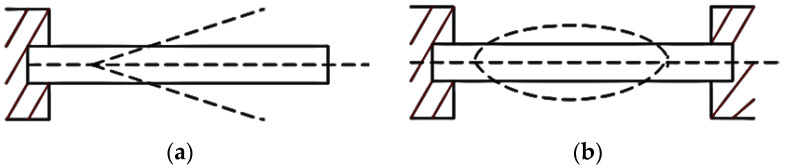
Two Kinds of Typical Vibration Acquisition Structure: (**a**) Cantilever Beam. (**b**) Simply Supported Beam.

The supporting model of the piezoelectric cantilever structure is shown in Figure 1a. Its main advantages are simple structure and convenient manufacture; at the same time, it can reduce the natural frequency of the cantilever beam, making the harvester more prone to resonance in the vibration source.

**Figure 2:**
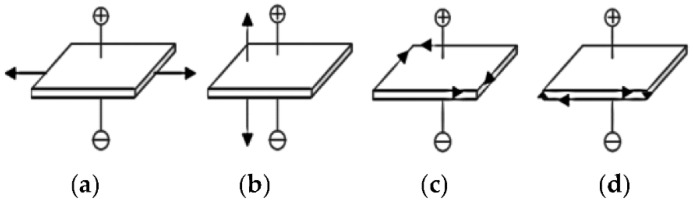
Vibration Mode of Piezoelectric Oscillator: (**a**) LE Model (**b**) TE Model (**c**) FS Model (**d**) TS Model.

There are four kinds of vibration modes, as shown in Figure 2, when piezoelectric materials are strained [19,20].

In Figure 2a, extension vibration of length perpendicular to the direction of the electric field is referred to as LE mode, and $d_{31}$ coupling mode usually adopts this vibration mode. When the piezoelectric cantilever vibrator is subjected to an external load, the piezoelectric vibrator will bend and deform, and the piezoelectric ceramics symmetrically bonded on the metal sheet will stretch on one side and shorten on the other; that is, its vibration mode is vertical to the electric axis, which belongs to LE model.

The piezoelectric equation describes the relationship between elastic and electrical variables of piezoelectric crystals. It characterizes the electromechanical coupling relationship. Because the mechanical boundary conditions are divided into mechanical freedom and mechanical clamping, and there are two kinds of electrical boundary conditions, electrical short and electrical open circuits, four kinds of piezoelectric equations are obtained. The second kind of piezoelectric equation, that is, Formula (1), corresponds to the second kind of boundary condition: mechanical clamping and an electrical short circuit [18].

(1) $$\left[\begin{array}{c} D\\ T \end{array}\right]=\left[\begin{array}{cc} \varepsilon^{S} & e\\ -e_{t} & c^{E} \end{array}\right]\left[\begin{array}{c} E\\ S \end{array}\right]$$

where *D*—Dielectric displacement; *T*—stress; *S*—strain; *E*—electric field intensity; *e*—piezoelectric strain coefficient; $e_{t}$—piezoelectric stress coefficient, the transpose of *e*; $C^{E}$—elastic stiffness constant; and $\varepsilon^{s}$—clamping permittivity.

In vibration mechanics analysis, the spring-mass-damping model can be used to describe the vibrational model of a single-degree-of-freedom system caused by external excitation [21]. When the external vibrational excitation $Z_{g}=Z_{0}\;sin\omega t$ acts on the system, forced vibration occurs in the system. From Newton’s second law, the second-order differential equation of the system can be expressed as follows [22]:

(2) $$M\ddot{Z}(t)+d\dot{Z}(t)+kZ(t)=-M{\ddot{Z}}_{g}(t)$$

where *M* is the equivalent mass of the whole system, $Z_{g}$ is the displacement of the ambient vibration, and *Z* is the displacement of M relative to $Z_{g}$. *k* is the elasticity coefficient, and *d* is the damping coefficient. The dynamic equation of electromechanical coupling can be expressed as Formulas (3) and (4), and they are divided into two parts: mechanical and electrical [23].

(3) $$G=L\ddot{S}+R\dot{S}+\frac{S}{C}+nV_{P}$$

(4) $$I_{P}=C_{P}{\dot{V}}_{P}$$

where *G* is the external vibrational excitation, *S* is the strain of the piezoelectric material, the equivalent capacitance *C* denotes the mechanical stiffness factor, *n* is the coupling coefficient, the equivalent inductance *L* represents the mass or inertia part, and the equivalent resistance *R* represents the loss caused by mechanical damping. $C_{p}$ is the intrinsic capacitance of the piezoelectric material and represents the electrical characteristics in the process of excitation, and $V_{p}$ represents the voltage at both ends of the piezoelectric sheet. Its direct influencing factor is mechanical stress σ. $I_{P}$ represents the current at both ends of the piezoelectric sheet, which is directly affected by the mechanical strain rate $\dot{S}$. From the above dynamic equation, the VEH based on a piezoelectric material could be simplified as a capacitive voltage source or current source in the circuit analysis. The simplified equivalent circuit model is often used in systems with less force-electric coupling [24].

## 3. Design and Test of Piezoelectric Vibration Energy Conversion Power Supply Scheme

### 3.1. Fabrication and Testing of a Vibration Energy Harvester Based on a PVDF Piezoelectric Thin Film

**Figure 3:**
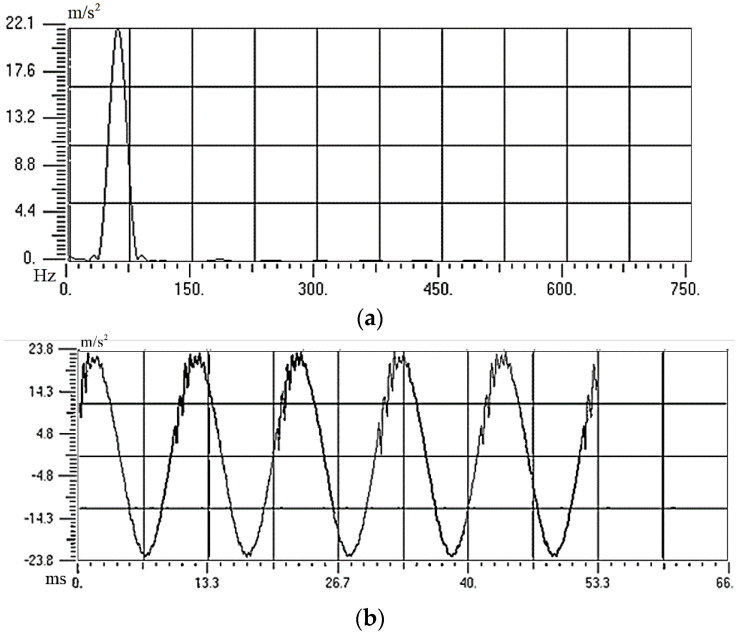
Vibration Signal of the Vibration Motor: (**a**) Amplitude Spectrum; (**b**) Acceleration Amplitude of the Time Domain.

Firstly, an FF-130SH three-speed vibration motor was adjusted to 4500 r/min. Then the vibration signal of the motor was measured and indicated in Figure 3. Motor-generated vibration excitation with a frequency of 75 Hz and acceleration amplitude of 2.198 g was used to simulate the surroundings of low-frequency vibrations.

**Figure 4:**
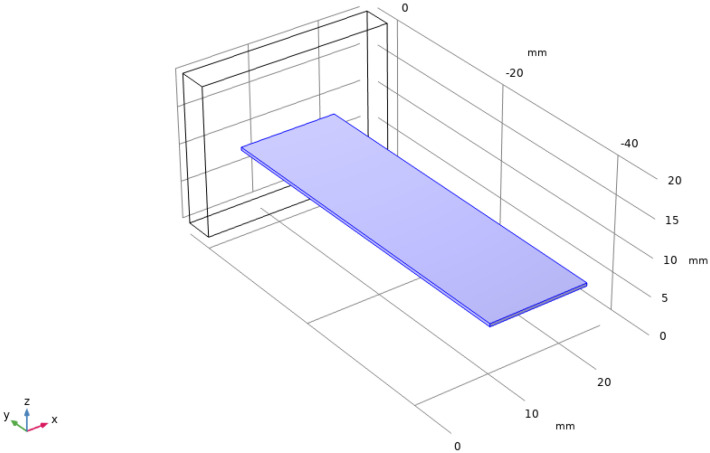
COMSOL 3D Model of the PVDF Piezo-film Vibration Energy Harvester Based on Motor-generated Vibration Signals.

Based on the theoretical analysis method in Section 2, a vibration energy harvester based on PVDF piezo-film was designed and modeled in COMSOL Multiphysics software, as shown in Figure 4. Then, the main body’s mechanical and electrical parameters of material input a parameter matrix in the software simulator are shown in Table 1. The mechanical clamping mode of the main body is set as single-end fixation, the upper and lower surfaces of the main body are output as electrodes, and corresponds to the LE vibration model and simulated boundary condition: mechanical clamping and an electrical short circuit. The vibrational incentive sources are set according to the parameters of the motor testing signal. When the cantilever beam of PVDF material reaches the first order resonance under this vibration excitation, the system frequency response solver in the software output with the dimensions of the main structure is 46.4 × 13.21 × 0.4 mm^3^.

**Figure 5:**
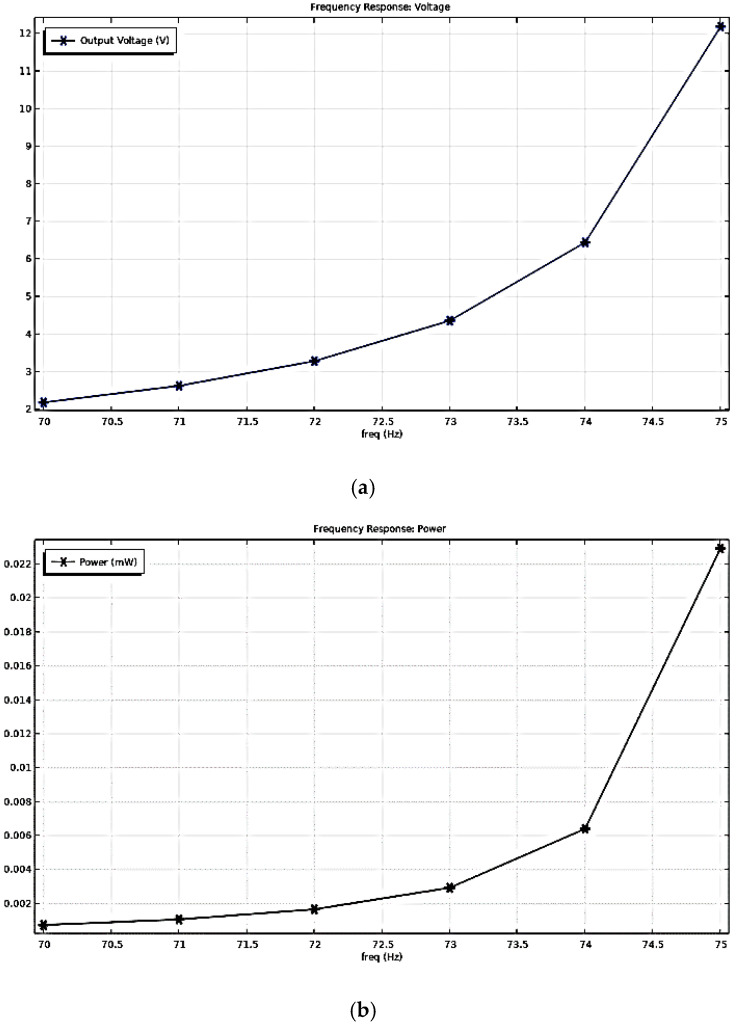
Simulation Results of the Harvester under the Motor Vibration Excitation: (**a**) Frequency Response of the Output Voltage; (**b**) Frequency Response of the Output Power.

When applying a body load to this model with the same vibration parameters as the motor, i.e., a uniformly distributed loading stress that production from a modulator in software by the vibration sources with a frequency of 75 Hz and acceleration amplitude of 2.198 g, the output voltage and power at the harmonic response was obtained, as shown in Figure 5. Under the resonance frequency of 75 Hz, the output voltage and power of the harvester model were 12.67 V and 23 µW, respectively.

So, we implemented physical fabrication based on this model. To reduce the fabrication difficulty, cost, and size, we utilized two 200 µm thick piezoelectric PVDF polymers that were synthesized and molded as raw materials and cut them into a film of the dimensions of 46.4 × 13.21 × 0.2 mm^3^. The main performance parameters of selected PVDF materials are shown in the first column of Table 1.

**Figure 6:**
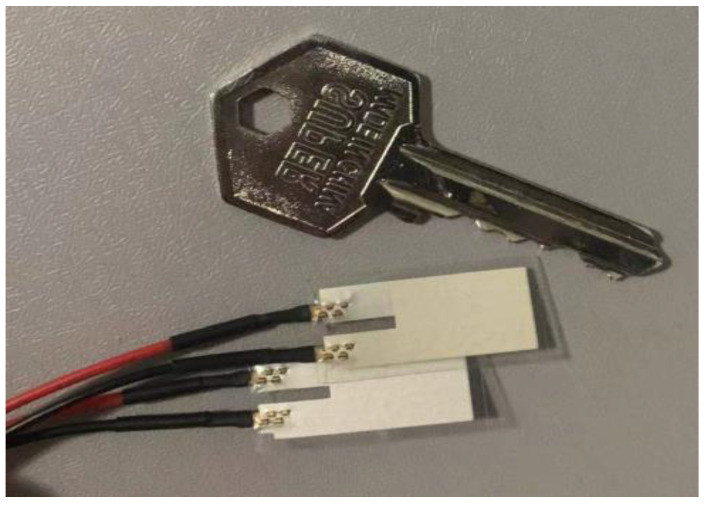
Photograph of Piezoelectric Thin Film.

Then, the films were embedded in screen-printed silver ink electrodes, laminated to a 125 µm polyester substrate, fitted with two crimped contacts, and packaged in a flexible plastic cover. The electrode of the upper and lower surfaces is led out by copper-core DuPont wires. The physical product of the piezoelectric thin film manufactured by us is shown in Figure 6. When in use, it can be fixed either at one end or by lamination.

In Table 1, the strength of the degree of conversion of energy is expressed by the coupling coefficient *k*, whose value is also related to the degree of polarization. This coefficient reflects the properties of the piezoelectric material and characterizes the percentage efficiency of the conversion of the input energy to the output energy. The first number in the subscript refers to the direction of the electric field, and the second number to the direction of the stress or strain. When the piezoelectric body is under constant stress, *d* is the polarization generated per unit of mechanical stress applied to a piezoelectric material. When the piezoelectric body is subjected to constant potential displacement, *e* is defined as the ratio of a component of the strain with respect to a component of the electrical field intensity.

The Poisson’s ratio only reflects the mutual influence of material deformation in different directions. The larger the Young’s modulus is, the stronger the material’s resistance to deformation is, and it is not easy to deform. The larger the Relative permittivity is, the stronger the ability to bind the charge is, and the larger the intrinsic capacitance is, the slower the discharge is. The Piezoelectric voltage constant is the electric field generated by a piezoelectric material per unit of mechanical stress applied. The Dielectric loss is defined as the current fracture rate during polarization. Additionally, The Density, Volume resistivity, Mechanical durability, and the Curie point represent lightness, life, resistivity, and thermal stability, respectively.

It can be seen from the data in Table 1 that the lightness, flexibility, life, and output voltage of PVDF are better than those of the other two types of materials, i.e., high mechanical properties. However, in terms of coupling coefficient, piezoelectric charge coefficient, relative permittivity, and other electric parameters, PZT and composite materials are better; that is, VEH based on PVDF materials is still a weak point in terms of energy conversion and output characteristics.

**Figure 7:**
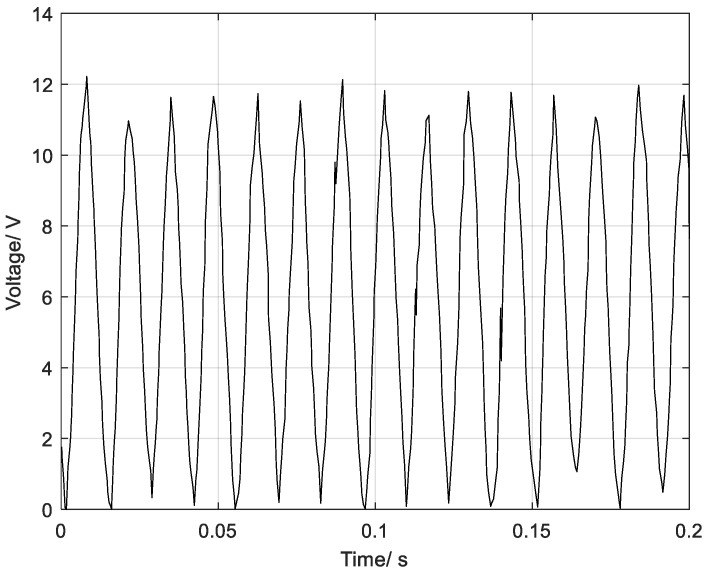
Rectifier Output Wave for the Piezoelectric Film under Motor Excitation.

First, the VEH based on a piezoelectric thin film was stuck on the motor shell to capture the vibration energy and measured its output with a multimeter and oscilloscope at the same time. In Figure 7, the oscilloscope shows the relationship between the output open voltage of the rectifier and time. The output peak voltage was approximately 12 V. Additionally, the output short-circuit AC current measured was 0.061 mA.

**Figure 8:**
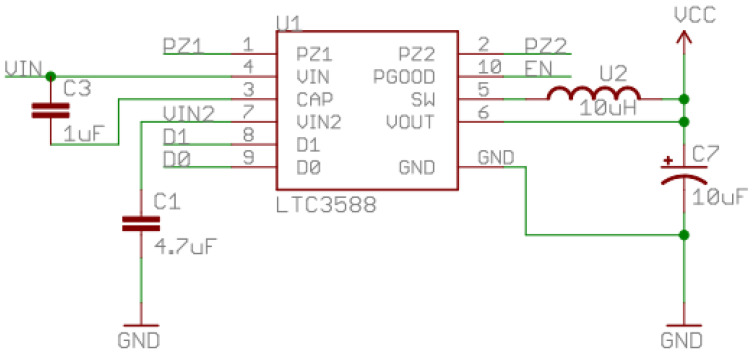
The Diagram of the Testbench.

Then, a vibration energy harvesting circuit based on the LTC3588-1 power management chip was built according to the peripheral circuit of Figure 8. The electrode output of the VEH is connected to the pz1/2 pins, and the VIN pin is connected to an energy storage element. The remaining capacitors and inductors are designed to trigger internal logic control [25].

To reduce the leakage rate and increase the charging rate and the withstand voltage value of the energy storage element, a 22 V-0.022 F ultracapacitor bank was used for storing energy. The charging current of the ultracapacitor decreased nonlinearly with the charging process, and the range was approximately 1~15 μA. The average rising rate of the charging voltage of the ultracapacitor was 0.1 mV/2 s. The average output power and maximum output power during charging were 20 μW and 26 μW, respectively. When the vibration stopped, no leakage was found in the ultracapacitor. In the actual test, it took 3 h to charge the ultracapacitor to 5 V with this output power.

Although the output voltage and power of the harvester meet the design expectation under the vibration motor as vibration excitation, the rate of voltage rise of the ultracapacitor is still very low. The charging time required to reach the input level of the rear circuit is very long. Obviously, for an ultracapacitor with a small capacity of 0.022 F, such a charging rate is not satisfactory for meeting the requirements of most applications.

### 3.2. Improved Design of the Vibration Energy Conversion Power Supply Using a Multiple Piezo-Film Structure

**Figure 9:**
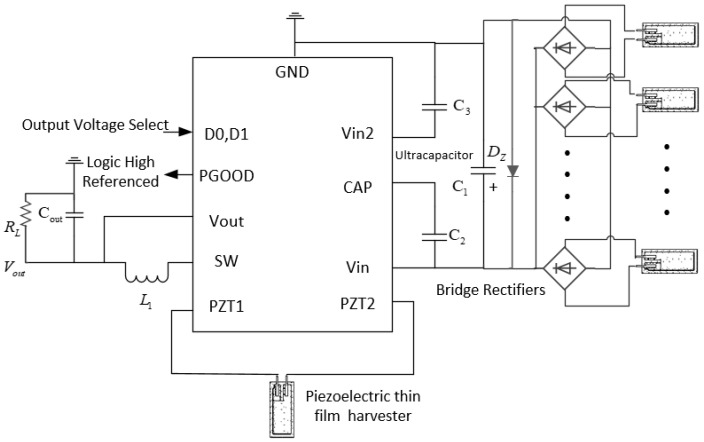
A Circuit for the Control of Multiple Current Sources.

PVDF is an ideal candidate material in the electrical and electronic fields because of its mature process, low cost, high flexibility, mechanical durability, thermal stability, and corrosion resistance. However, in light of Table 1, compared with inorganic or compound piezoelectric materials, the dielectric constant and piezoelectric constant of PVDF are much lower [13,26]. The test results in the previous section reflect these shortcomings of PVDF materials. To further improve the charging rate and the energy conversion efficiency of VEHs based on PVDF, we propose a vibration energy harvesting structure based on a PVDF thin film planar array to replace a single film and design an electric energy regulation circuit for paralleling multiple current sources input. From the discussion in Sections II, the piezoelectric vibration energy harvester can be equivalent to an AC power supply. Therefore, Figure 9 shows the multi-source charging circuit composed of multiple films.

Connecting multiple power sources in parallel can increase the charging current and vibration energy conversion efficiency. Additionally, before setting up this circuit, the number of thin films should be determined according to the requirements of an actual application.

**Figure 10:**
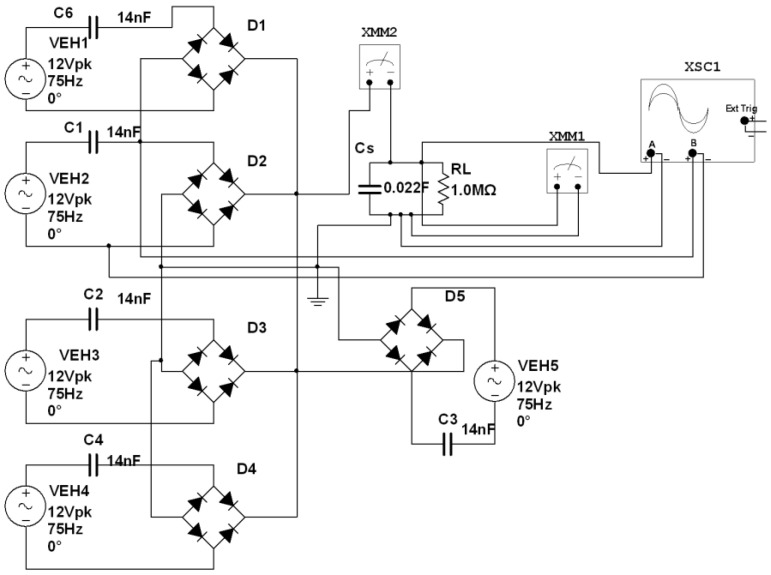
Diagram of the Charging Circuit of Parallel Multi-sources in Simulation Software.

Based on the motor’s vibration parameters, the output electrical parameters’ characteristics, and the elastic characteristics of the piezoelectric film, the parameters of the equivalent AC voltage source are determined: $V_{p}$ = 12 V, *ƒ* = 75 Hz, $C_{p}\;$= 14 nF, and *φ* = 0. When *N* = 1, 2, 3, 4, and 5, the charging process of parallel multi-sources is simulated by the Multisim simulation software. The simulation process is shown in Figure 10.

**Figure 11:**
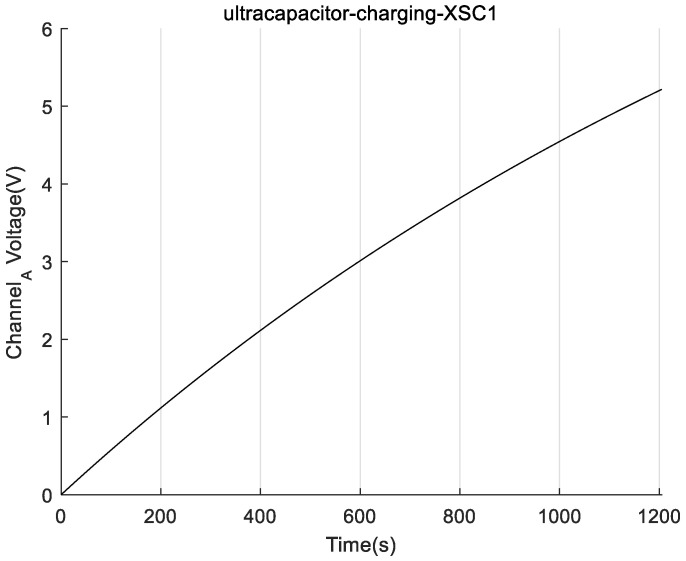
Capacitor Charging Voltage for a Five Piezoelectric Film Parallel Equivalent Simulation Circuit.

**Figure 12:**
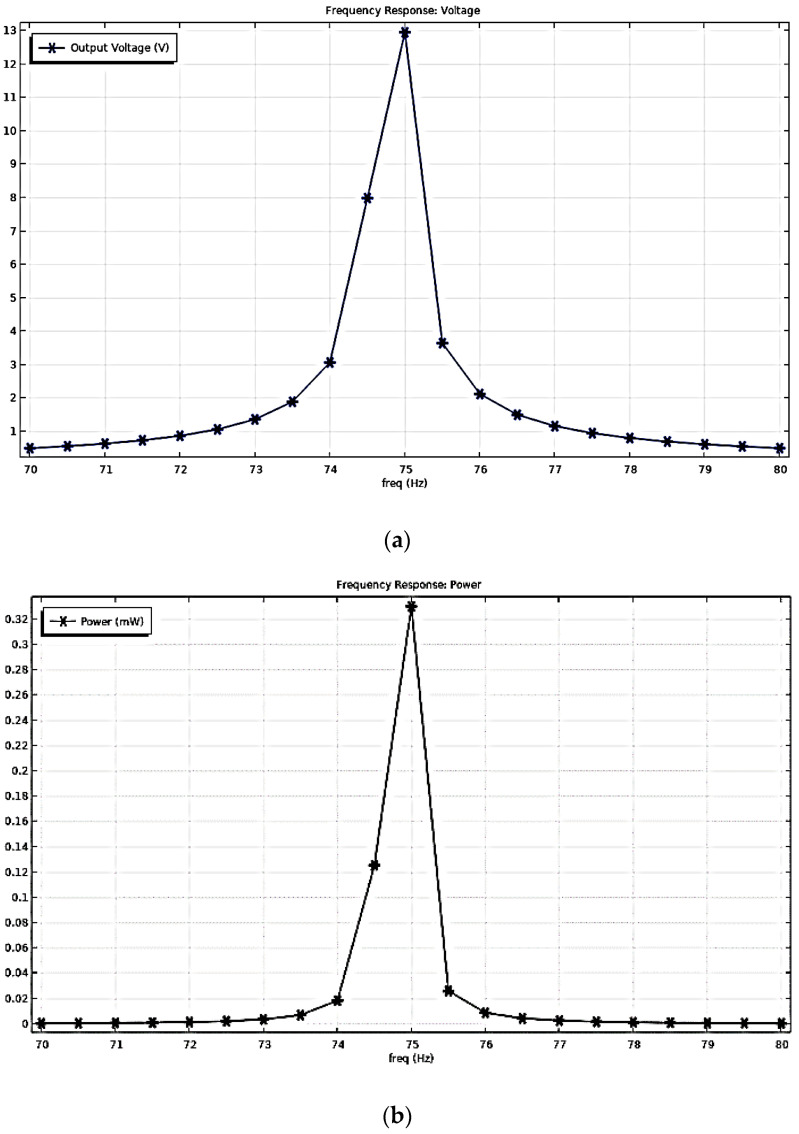
Simulation Results of the Harvester of the Five Piezo-films Planar Array under the Motor Vibration Excitation: (**a**) Frequency Response of the Output Voltage; (**b**) Frequency Response of the Output Power.

During the simulation process, the output voltage of each film, the charging voltage and the charging current of the ultracapacitor, and the times for ultracapacitor charging to 3.3 V and 5 V were recorded. The simulation results of different quantities of piezo films in parallel to generate electricity are shown in Table 2. Moreover, the simulated voltage waveform for capacitor charging when the *N* is equal to 5 is plotted in Figure 11.

(5) $$\{\begin{array}{c} P_{av}=\frac{W_{c}}{t_{c}}=\frac{0.5*C*\Delta U_{c}^{2}}{t_{c}}=\frac{0.5\times 0.022\times 5^{2}}{19.87\times 60}=0.231mW\\ P_{\max}=\max{\left\{U_{c}*I_{c}\right\}}_{t\in t_{c}}=2.647V*140.914\mu A=0.373mW \end{array}$$

where $I_{C}^{’}$ is the average charging current during the time for ultracapacitor charging to 3.3 V and $I_{C}$ is the average charging current during the ultracapacitor charging from 3.3 V to 5 V. According to the simulation data of Table 2 and Figure 12, the average and maximum charging power of a single film from charging the empty capacitor to 5 V are 33 µW and 35 µW, respectively. Additionally, that of the five piezo films are, respectively, 0.231 mW and 0.373 mW, which is calculated from Equation (5). The parameters with the character “c” in the lower corner are those obtained during the simulation of charging to the capacitor.

Furthermore, the charging time of the ultracapacitor decreases with the number of films and the average charging current increases with the number of films. Meanwhile, when the number of parallel films, namely, *N*, doubles, the average charging current value is doubled, and the whole charging period is halved. Based on the simulation results of the equivalent circuit.

**Figure 13:**
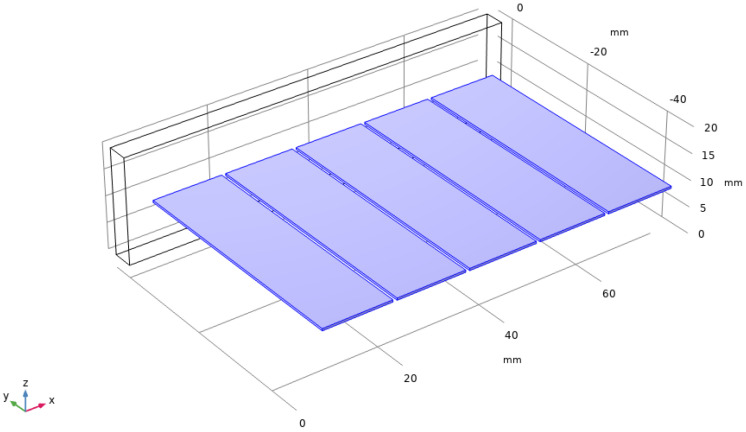
VEH Based on the Vibration−Capturing Structure of the Piezo-film Planar Array.

Similar to the theoretical analysis and modeling approaches for the single film harvester in Figure 4, a multi-piezoelectric film planar array-based vibration energy harvester was built in COMSOL, as shown in Figure 13. Above all, the mechanical input of each piezoelectric transducer unit in the array has been uniformly set when the model is established, and the input vibration signals are completely consistent. The output of each piezoelectric transducer unit is virtually electrically coupled, and they output power on the global variable impedance.

At the same time, the body load was applied to each harvester unit in this model with the same vibration parameters as the motor, and the simulated output voltage and power at the harmonic response of the different piezo-film array structures were recorded in Table 3. From the FEA (Finite Element Analysis) simulation results, it appears that the output power of the harvester increases approximately doubly as adding one to the number of films; in addition, the output voltage remains essentially constant because the body load on each film is exactly the same. This is, therefore, similar to the inference from the simulation results for the equivalent circuit model, except that deviations in the modeling approach process result in different data values.

The simulation results of the five piezo-films array structures were obtained, as shown in Figure 11. Under the resonance frequency of 75 Hz, the output voltage and power of the harvester model were 12.962 V and 0.339 mW, respectively. Compared to the results in Figure 7b, the output power of the five-piece films is 14.74 times greater than that of a single one. Therefore, this piezoelectric thin film array vibration-capturing structure can effectively increase the output power and energy conversion efficiency of the whole device.

### 3.3. Fabrication and Experimental Testing of the Vibration Energy Conversion Power Supply Using a Multiple Piezo Film Structure

**Figure 14:**
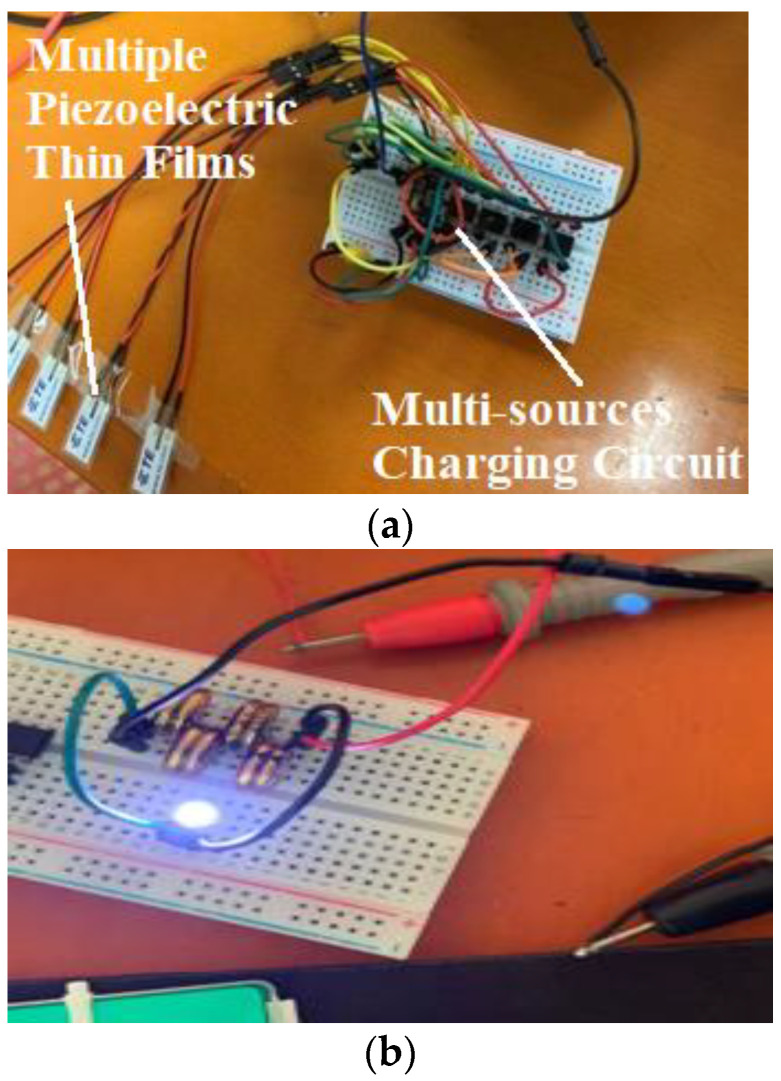
Physical Product and the Energy Storage Test of Multi-source Charging Circuit: (**a**) Physical Product and Test Preparation of Multi-sources Charging Circuit; (**b**) Electric Circuit of Harvesting with Successfully Lit LED.

Taking *N* = 5 as the test sample, the physical circuit that was built according to Figure 9 is shown in Figure 14a.

**Figure 15:**
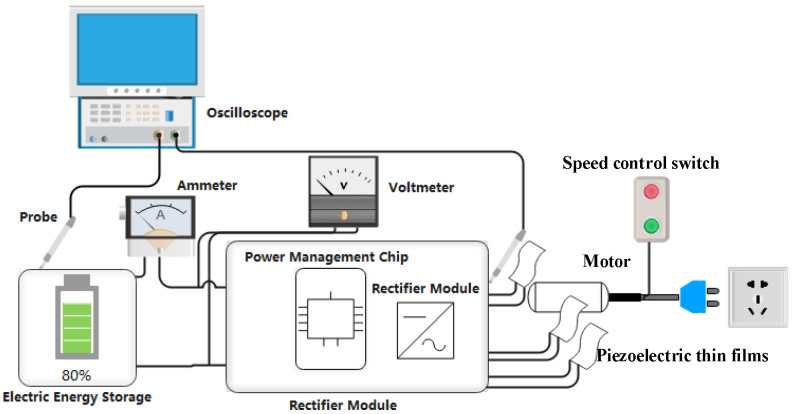
Equipment Setup of Vibration Energy Harvesting Testing.

In order for the vibration amplitude of each piezo film to be the same and the time difference of sensing vibration to be the shortest, five pieces of piezoelectric thin film stuck to the surface of the motor housing, side by side, without overlapping. The equipment setup for the testing of vibration energy harvesting is shown in Figure 15.

**Figure 16:**
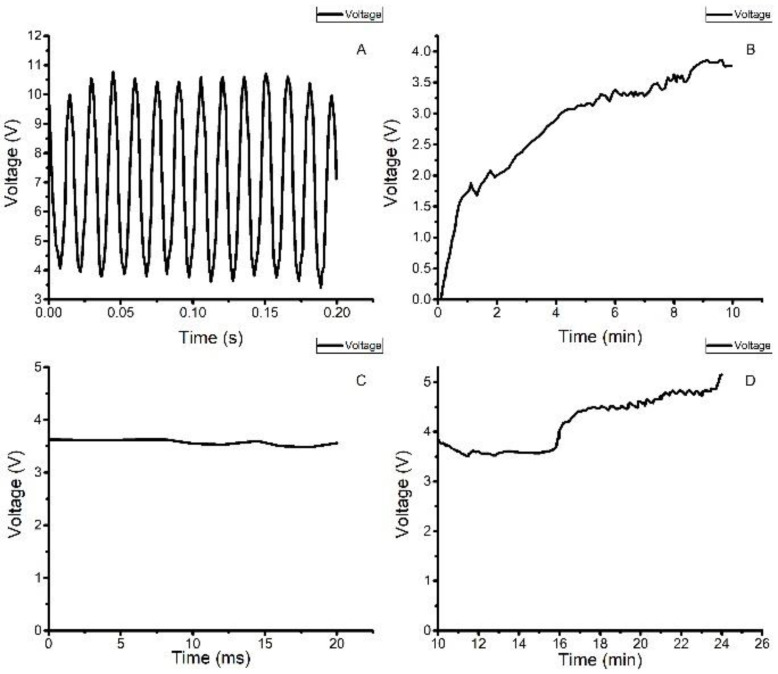
Voltage Waveform in Charging Experiment: (**A**) Rectifier Output Wave; (**B**) The Voltage Waveform of the Supercapacitor in 0–3.5 V; (**C**) The Output Voltage of the Vibration Energy Harvesting Circuit; (**D**) The Voltage Waveform of the Supercapacitor for 3.5–5 V.

The output AC voltage of each film was rectified by a DB107 IC, and the terminals of the rectifiers were input in parallel to the VIN pin of the LTC3588-1 power management chip. Figure 16A illustrates the rectifier output voltage wave of the DB107. The peak value of the output voltage is up to 10.4 V, and the DC effective value is 5.58 V. Compared with the rectification waveform of Figure 7, due to the superposition of the output voltage out of phase, the amplitude of the output voltage is smaller and contains a DC bias voltage of 4 V.

Figure 16B,D show the charging voltage waveform of the supercapacitor bank. Before the ultracapacitor’s charging voltage reaches 3.5 V, the average charging current is 0.161 mA, and the voltage of the ultracapacitor increases at an average rate of 0.1 V/12 s. When the ultracapacitor charging voltage is in the range of 3.5 V to 5 V, the average charging current is 0.053 mA, and the ultracapacitor’s voltage increases at an average rate of 0.1 V/22 s.

Figure 16D shows that there is a stable period of charging voltage of approximately 4 min, which is caused by the fatigue effect due to the long-term excitation of the PVDF thin film [27]. After a period of time, the internal conversion mechanism of the piezoelectric material recovers and continues to charge the ultracapacitor bank until the final voltage of 5 V.

Finally, it took approximately 24 min to complete the whole charging process. We measured the standard output voltage of 3.6 V at the output pin of the LTC3588-1 chip, and the output waveform is shown in Figure 16C. With the same calculations as in Equation (5), the average output power of the VEH was approximately 0.191 mW, which is 9.55 times higher than that of a single film; the maximum charging power was 0.304 mW, which is 11.69 times higher than that of a single film. The electrical energy stored can be calculated from Equation (6).

(6) $$W_{C}=\frac{C*\Delta U^{2}}{2}=0.5*0.022*5^{2}J=0.275J$$

As shown in Figure 14b, the energy stored in the supercapacitor bank could light a 60-mW LED, which is in a high brightness state for approximately 5 s. According to our investigations and discoveries, the power consumption of micro-electronic products on the market is mostly in the range of 10~10 mW. The power consumption of Bluetooth RF devices is mostly in the range of 0.3~3 mW when transmitting 10 bytes/s [28]. Therefore, the harvested power can provide a continuous working time of the Bluetooth RF device for at least:

(7) $$\{\begin{array}{c} W_{c}^{’}=\frac{C*\Delta U^{2}}{2}=0.5\times 0.022\times {\left(5-1.8\right)}^{2}J=0.11264J\\ \Delta t_{RF\_\min}=\frac{W_{c}^{’}}{P_{\max}}=\frac{0.11264J}{3mW}\approx 37s \end{array}$$

It can be seen from the test results that the rectified voltage, charging time, and output power deviate from the theoretical simulation results. The reason is that the simulation circuit and simulation structure are in the ideal synchronous matching state of the power supply output, so there is no mutual phase staggering reduction of the DC quantity. However, in an actual circuit or vibration structure, because these parallel films cannot be fully synchronized during vibrational sensing, the capacitance only takes their maximum DC voltage as the final charging voltage, and the charging currents are also weakened. In addition, the output power may be affected by the fatigue effect of the material. Hence, the simulation effect of two theoretical models cannot be fully achieved in the test; however, these deviations are acceptable and in line with the theoretical expectation. In summary, after we implemented the vibration energy harvester based on the piezo-film planar array, the energy conversion efficiency, output power, and charging rate of the whole device made a great improvement. From the experimental results, the power supply proposed in this paper has met the practical requirements of most low-power electronic devices.

## 4. Conclusions

To improve the charge output and conversion efficiency of VEH based on PVDF materials, the planar array vibrational structure of multiple PVDF piezoelectric thin films was proposed. In addition, an energy harvesting circuit based on an LTC3588-1 power management chip with a multi-source parallel rectifier circuit topology was designed to improve the output power and charging rate. We used both of them to constitute a vibration energy conversion power supply; meanwhile, it was validated by simulations of structural dynamics and equivalent circuits. The simulation results show that as the number of films increases, the output voltage does not change much, while the output power doubles or increases even higher. For example, in both simulation models, when the number of films in the array group is 5, the maximum power output is expanded by more than 10 times compared to a single film. The vibration energy harvesting test was completed in a vibration environment with an acceleration of 2.198 g and a vibration frequency of 75 Hz. The power supply quickly charged the 22 V-0.022 F ultracapacitor bank to 5 V in 24 min. The maximum open circuit voltage and output power, respectively, were 10.4 V and 0.304 mW. This maximum charging power was 11.69 times higher than that of a single film. By comparing the experimental test results obtained with the theoretical values simulated, the rationality of the theoretical design is verified. The harvested electricity could be successfully converted to a 3.6 V standard supply voltage by the energy harvesting circuit and maintained a 60-mW LED at stable high brightness for 5 s or kept a Bluetooth RF device working continuously for at least 37 s. In terms of output power, charging rate, output voltage, and loading capacity, the vibration energy conversion power supply has reached the standard of practicality and exceeds batteries that are not able to keep up with the demand for IoT devices on certain occasions.

## Figures and Tables

**Table 1:** Comparison of main parameters of common piezoelectric materials.

	Model	PVDF	PZT	MFC
Parameter				
Density/kg·m^−3^		1780	7500	5440
Poisson’s ratio		0.3	0.36	0.312
Young’s modulus/GPa		2.5	76.5	5.52
Piezoelectric charge coefficient d_33_/pC·N^−1^		33	640	467
Coupling factor k_33_		0.14	0.76	0.339
Piezoelectric voltage constants g_33_/Vm·N^−1^		0.34	0.02	0.275
Relative permittivity Ɛ_r3_^T^		13.2	3400	1700
Dielectric loss tanδ%		8.9	1.8	2
Volume resistivity /Ω·m^−1^		10 × 10^15^	10 × 10^11^	10 × 10^13^
Mechanical durability/times		2 × 10^11^	3.5 × 10^8^	1.0 × 10^10^
Curie point Tc/°C		170	300	176

**Table 2:** Simulation results of a multi-power charging circuit.

*N*	$I_{C}^{’}(\mu A)$	$I_{C}\;(\mu A)$	$\Delta t_{0\to 3.3V\;}(\min)$	$\Delta t_{0\to 5V}\;(\min)$
1	39.22	11.44	35.64	138.40
2	78.10	24.46	19.84	69.30
3	113.53	34.12	15.74	42.50
4	155.14	46.62	12.34	33.50
5	193.17	58.23	11.16	19.87

**Table 3:** Simulation results of a multi-piezo film harvester.

*N*	$V_{oc}\;(V)$	$P_{max}\;(\mathrm{mW})$
1	12.671	0.023
2	12.732	0.058
3	12.772	0.137
4	12.827	0.226
5	12.962	0.339

## Data Availability

Not applicable.
